# Association between Dairy Consumption and Psychological Symptoms: Evidence from a Cross-Sectional Study of College Students in the Yangtze River Delta Region of China

**DOI:** 10.3390/ijerph20043261

**Published:** 2023-02-13

**Authors:** Zhimin Zhao, Ruibao Cai, Yongxing Zhao, Yanyan Hu, Jingzhi Liu, Minghao Wu

**Affiliations:** 1 School of Physical Education, Chizhou University, Chizhou 247000, China; 2 Sports Health Promotion Center, Chizhou University, Chizhou 247000, China; 3 Research Department of Physical Education, Xinjiang Institute of Engineering, Urumqi 830023, China

**Keywords:** dairy consumption, psychological symptoms, cross-sectional studies, college students, Yangtze River Delta region

## Abstract

Background: Assessing the dairy consumption and psychological symptoms of Chinese college students as a reference for the mental health of Chinese college students. Methods: A three-stage stratified whole-group sampling method was used to investigate dairy consumption and psychological symptoms among 5904 (2554 male students, accounting for 43.3% of the sample) college students in the Yangtze River Delta region. The mean age of the subjects was 20.13 ± 1.24 years. Psychological symptoms were surveyed using the Brief Questionnaire for the Assessment of Adolescent Mental Health. The detection rates of emotional problems, behavioral symptoms, social adaptation difficulties and psychological symptoms among college students with different dairy consumption habits were analyzed using chi-square tests. The association between dairy consumption and psychological symptoms was assessed using a logistic regression model. Results: College students from the “Yangtze River Delta” region of China participated in the study, of which 1022 (17.31%) had psychological symptoms. The proportions of participants with dairy consumption of ≤2 times/week, 3–5 times/week, and ≥6 times/week were 25.68%, 42.09%, and 32.23%, respectively. Using dairy consumption ≥6 times/week as a reference, multifactor logistic regression analysis showed that college students with dairy consumption ≤2 times/week (OR = 1.42, 95% CI: 1.18, 1.71) were at higher risk of psychological symptoms (*p* < 0.001). Conclusion: During the COVID-19 pandemic, Chinese college students with lower dairy consumption exhibited higher detection rates of psychological symptoms. Dairy consumption was negatively associated with the occurrence of psychological symptoms. Our study provides a basis for mental health education and increasing knowledge about nutrition among Chinese college students.

## 1. Introduction

Milk, milk powder, cheese, and other dairy products are rich in nutrients, such as calcium, protein, potassium, and phosphorus [1]. Dairy products are an important part of the human diet [2], providing between 9.0 and 12.0% of an individual’s energy needs [3]. For example, in Western countries, up to two-thirds of the population’s calcium intake comes from dairy products, demonstrating the importance of dairy products for bone health [4]. Recent studies suggest that the consumption of dairy products appears to have beneficial effects on building muscle, cardiovascular disease risk reduction, prevention of tooth decay, diabetes, cancer, and obesity [2,5]. Epidemiologically-based studies have also demonstrated a significant negative association between the intake of dairy products (especially the low-fat variety) and the prevalence of metabolic syndromes, such as hypertension, dyslipidemia, and abdominal obesity [6]. Dairy consumption has been associated with a 13% reduction in the risk of all-cause mortality (RR: 0.87, 95% CI: 0.77, 0.98) [7]. Given the importance of dairy products for health, dairy consumption has increased significantly in developed countries [8], and the average intake of dairy products by Chinese residents increased from 14.9 g/d in 1992 to 24.7 g/d in 2012 [9].

The World Health Organization describes mental health as the foundation of human health, and mental health disorders can seriously affect physical health, personal well-being, and daily life [10,11,12]. According to the World Health Organization, as many as 80,000 people worldwide die by suicide each year because of depression among young people aged from 15–29 [13]. However, psychological symptoms such as depression, anxiety, and stress are prevalent in the college student population [14], and during the coronavirus disease of 2019 (COVID-19) pandemic, approximately 40.0% of Chinese college students experienced anxiety symptoms [15]. Some studies have shown that the rates of depression and anxiety symptoms among college students have increased each year over approximately the past decade, from 15.6% [16] in 2005 to 31.0% in 2018, representing an increase of 15.0% [17].

A growing number of studies have suggested that healthy eating habits may reduce the risk of developing mental illness [18,19]. For example, the intake of dairy products (skim milk) in adults was negatively associated with depressive symptoms, whereas whole milk and low-calcium dairy products were positively associated with symptoms of depression and insomnia in adults [20,21]. Clinical trials conducted in various countries have reported that the consumption of probiotic-containing dairy products or dairy products in general by patients with depression is associated with a reduction in depressive symptoms [22,23,24]. However, a prospective analysis of Italian adults found no association between depressive symptoms and dairy intake [25]. This suggests that the relationship between dairy products and mental health warrants further investigation.

The above findings are from studies of adults, and there is limited research on college student populations. A study from the Azores showed that the consumption of fermented dairy products had a positive effect on reducing anxiety in young college students [26]. In addition, probiotics have been reported to improve panic anxiety, neurophysiological anxiety, negative emotions, and worry, and to increase the regulation of negative emotions in college students [27]. To the best of our knowledge, no previous studies have examined the relationship between dairy product consumption and mental health among Chinese college students.

College students are undertaking an important period of transition from adolescence to adulthood, and their dietary habits and psychological health are closely related to their later life development. To this end, this study investigated dairy consumption and psychological symptoms among 5904 college students (2554 males, 43.3%) in the Yangtze River Delta region of China. The aim was to assess the relationship between dairy consumption and psychological symptoms among Chinese college students, with the aim of providing a reference and basis for the healthy development of college students.

## 2. Materials and Methods

### 2.1. Participants

To ensure the representativeness of the data, a stratified whole-group sampling method was used to select the sample size for this study. First, we selected Hefei, Shanghai, Suzhou, and Hangzhou in the Yangtze River Delta region as the survey cities. Second, we selected two colleges in each city as the surveyed schools. Third, electronic questionnaires were distributed in each college using online publicity and posters. Finally, a total of 5997 college students were surveyed from eight colleges. After excluding students with incomplete demographic information, a total of 5904 valid data sets were obtained. The effective recovery rate of the questionnaire was 98.45%. There were 2554 male students, accounting for 43.3% of the sample. The mean age of the subjects was 20.13 ± 1.24 years.

In the current study, the survey was conducted after college students gave written informed consent. The survey was implemented after obtaining approval from the Human Ethics Committee of Chizhou University (202104090).

### 2.2. Data Collection Process

Before the test, we introduced the test content of the project and the purpose and significance of the study to the school in detail. The participants were informed of the purpose and significance of the study before the survey. The participants filled out the questionnaire by scanning the QR code using their cell phones. To ensure the authenticity of the data, the study participants were given sufficient time to fill out the relevant contents independently and were given consultation and answers to questions in the process of filling out the questionnaire via WeChat or telephone.

### 2.3. Dairy Consumption

Dairy consumption data were obtained by conducting a questionnaire survey on the subjects. The specific questions were as follows: How many times have you consumed dairy in the past week? For example, liquid milk (yogurt, etc.), dairy powder (whole milk powder, skim milk powder, etc.), milk fat, cheese, other dairy products, etc. For the convenience of data analysis, our study divided dairy consumption into three groups: ≤2 times/week, 3–5 times/week, and ≥6 times/week.

### 2.4. Psychological Symptoms

We assessed college students’ psychological symptoms using the Brief Questionnaire for the Assessment of Adolescent Mental Health, which was divided into three dimensions of emotional problems (seven questions), behavioral symptoms (four questions), and social adaptation difficulties (four questions), with a total of fifteen questions, and the total score of all questions was the total score of psychological symptoms. The questionnaire has been widely adopted among Chinese university students and has good reliability and validity. Each question was filled in according to the participants’ actual situation, and each question had six options, in the order of (1) “lasted more than 3 months”, (2) “lasted more than 2 months”, (3) “lasted more than 1 month”, (4) “lasted more than 2 weeks”, (5) “lasted more than 1 week”, and (6) “none or less than 1 week”; selecting (1)–(3) scored 1 point, selecting (4)–(6) scored 0 points. When the score of emotional problems dimension was ≥4, the presence of emotional problems was judged; when the score of character problems dimension was ≥1, the presence of social use difficulties was judged; when the score of social adaptation difficulties was ≥2, the positive result of this dimension was indicated. The presence of psychological symptoms was determined when the total score of the three dimensions was ≥7.

### 2.5. Covariates

The investigation of covariates in our study included age, sex, socioeconomic status (SES), screen time, body mass index (BMI), and moderate and vigorous physical activity (MVPA). Age was calculated using the participants’ last birthday, and SES was judged by the education, occupation, and income of college students’ parents, which were divided into low (<25th percentile), medium (25–75th percentile), and high (>75th percentile). Height (m) and weight (kg) were measured for college students according to the testing requirements of the National Physical Fitness Standards for Students in China, with an accuracy of 0.1 cm and 0.1 kg, respectively. BMI was calculated as weight (kg)/height(m)^2^. MVPA time per day for college students was calculated by investigating the average daily frequency and time of moderate and vigorous physical activity of the participants for the previous week using a questionnaire.

### 2.6. Statistical Analysis

Categorical data were expressed by percentage and continuous data by mean ± standard deviation. The cardinality test was used to analyze the dairy consumption status of different groups. The continuous type variables in this study conformed to a normal distribution. Comparisons of continuous variables such as BMI and MVPA between different dairy consumption habits were performed by single factor variance. Logistic regression models were used to assess the association between dairy consumption and psychological symptoms. The Crude Model, Model 1 (controlling for age and SES), and Model 2 (controlling for screen time, sugar-sweetened beverages, sleep quality, BMI, and MVPA on the basis of Model 1) were used to analyze the association between college students’ dairy consumption and emotional symptoms, behavioral symptoms, social adaptation difficulties, and psychological symptoms, respectively, reporting the odds ratio and 95.0% confidence interval. Analysis was performed using SPSS 25.0 (SPSS Inc., Chicago, IL, USA) software with a two-sided test level of *α* = 0.05.

## 3. Results

This was a cross-sectional study in which we investigated the dairy consumption of 5904 (2554 males, 43.3%) college students during the COVID-19 pandemic. The results of the study showed that the proportions of college students that consumed dairy ≤2 times/week, 3–5 times/week, and ≥6 times/week were 25.68%, 42.09%, and 32.23%, respectively.

Our study showed that, in terms of count variables, the differences in dairy consumption detection rates among college students significantly differed by sex, SES, screen time, SSB consumption, and sleep quality (*χ*^2^ = 43.115, 87.779, 39.678, 87.275, 54.136, *p* < 0.001). In terms of continuous variables, the differences in dairy consumption detection rates of MVPA among college students were significant (*F* = 29.821, *p* < 0.001) (Table 1).

Our survey revealed that during the COVID-19 pandemic, the detection rate of psychological symptoms among college students in the Yangtze River Delta region was 17.31% (1022/5904). The detection rate of psychological symptoms for male students was 16.05% (410/2554) and for female students was 18.27% (612/3350); the differences in the detection rate of psychological symptoms between males and females was significant (*χ*^2^ = 4.969, *p* < 0.05).

Overall, the lowest detection rate of psychological symptoms was 13.8% for dairy consumption ≥6 times/week, followed by 16.8% for 3–5 times/week. The highest detection rate of psychological symptoms was 22.5% for individuals with dairy consumption of ≤2 times/week, and the difference was significant (*χ*^2^ = 45.062, *p* < 0.001). In terms of different dimensions, the trend of the magnitude of detection rates of emotional symptoms, behavioral symptoms, and social adaptation difficulties among college students was consistent with that for psychological symptoms, with ≤2 times/week (23.6%, 23.7%. 19.7%) exhibiting the highest percentage of college students, followed by 3–5 times/week and ≥6 times/week, which all showed significant differences (*χ*^2^ = 38.278, 40.611, 34.521, *p* < 0.001). In addition, the same trend was found between the males and females (Table 2).

After adjusting for relevant covariates, the Model 2 analysis showed that college students with dairy consumption ≤2 times/week (OR = 1.42, 95% CI: 1.18, 1.71), using dairy consumption ≥6 times/week as a reference, were at a higher risk of psychological symptoms (*p* < 0.001). Similarly, the risk of emotional symptoms (OR = 1.38, 95% CI: 1.08, 1.77) was higher among female students with dairy consumption ≤2 times/week compared with college students with dairy consumption ≥6 times/week (*p* < 0.001). Our findings suggest that dairy consumption was negatively associated with the occurrence of psychological symptoms among Chinese college students during the COVID-19 pandemic (Table 3).

## 4. Discussion

Dairy is closely related to human health, and the results of our study showed that the proportion of college students with dairy consumption ≥6 times/week was 32.23%, whereas the proportion of college students with dairy consumption ≤2 times/week was 25.68%. This result indicated that the proportion of college students with regular dairy consumption was generally low, which is consistent with data from the China Statistical Yearbook, 2021, published by the National Bureau of Statistics of China, but substantially different to the recommended amount of dietary nutrition guidelines for China in 2021 [9]. Although the living standards of Chinese residents have been improving, and the intake of dairy products has increased, the overall level is still low [9]. The proportion of dairy consumption among Chinese college students is higher than that of Chinese adults, and much lower than that of European and American countries. The results showed that 20.4% of Chinese adults consume dairy products at least once a week, and 68.5% of respondents reported they never or rarely consume dairy products [28]. Among college students in Azorean, 91.8% of female students and 98.8% of male students were reported to consume at least one serving of dairy products between two and four times per week [26]. The reason for this difference may be related to differences in the geographical dietary habits of the study population.

Our study showed that the detection rate of psychological symptoms among Chinese college students was 17.31%, which was higher than that reported in previous studies. A study conducted during the COVID-19 pandemic reported that the detection rate of psychological symptoms among Chinese college students was 8.10% [29]. A study prior to the COVID-19 pandemic reported that the detection rate of psychological problems among college students was 9.98% [30]. The studies indicated that the detection rate of mental health problems among Chinese college students ranged from approximately 45.0% to 47.8% [31] from the beginning of the COVID-19 pandemic to the present. The psychological symptom detection rates among college students decreased as COVID-19 was controlled, and the rates of lethality and severe illness decreased. During the COVID-19 pandemic, many colleges conducted substantial public health education and mental health education to promote students’ mental health, resulting in a gradual decrease in the detection rate of psychological symptoms among college students. The current study also revealed that the detection rate of psychological symptoms was slightly higher in female students (18.26%) compared with that in male students (16.05%). This difference could be explained by the stress reactivity model to some extent, which attributes this difference to the differences between males and females in reactivity under stress [32].

Many previous studies have shown that dietary habits and diet types have an effect on psychological symptoms. Our study showed that college students with dairy consumption ≥6 times/week had the lowest detection rate of psychological symptoms compared with those with ≤2 times/week, and less dairy consumption was associated with a higher detection rate of psychological symptoms. This result is consistent with the findings of previous studies [33]. Some previous studies have reported an inverse relationship between dairy consumption and anxiety [34], with skim milk and moderate dairy-based dessert intake being negatively associated with depressive symptoms, whereas whole milk was positively associated with depressive symptoms in adults [35]. However, some studies have reported that dairy consumption had no positive effect on mental health [36]. This situation suggests that more attention should be paid to the mental health of college students and that more in-depth research on the effects of dairy consumption on mental health is required.

The results of the current study revealed an association between college students’ dairy consumption and psychological symptoms, and college students with dairy consumption ≤2 times/week had the highest detection rate of psychological symptoms. Thus, dairy consumption was an important factor affecting college students’ mental health, possibly related to the combined effects of nutrients in dairy and intestinal microorganisms. Dairy products are rich in tryptophan, which is an important raw material for the production of 5-hydroxytryptamine (serotonin) in the body, and low serotonin levels are associated with higher anxiety [37,38]. The gut microbiome produced by the fermentation of dairy products in the intestine has been reported to play a positive role in depressive symptoms, anxiety, cognitive function, sleep, and brain function [39]. It has been reported that gut microbes can alleviate stress and depression-related symptoms by modulating brain function [40]. The intake of dairy products can increase blood tryptophan levels and improve sleep quality and mood, thus, promoting mental health among college students.

The current research has several advantages. On the one hand, the sample size of our study was relatively large and representative. The sample was selected from several groups of college students with different majors in the Yangtze River Delta region, which has significant representativeness. In addition, our study effectively adjusted for additional factors that could potentially affect psychological symptoms, such as SES, screen time, sugar-sweetened beverage consumption, sleep quality, MVPA, and body mass index. On this basis, we further analyzed the association between dairy consumption and psychological symptoms among Chinese college students so that the results were more reliable. However, the current study has some limitations. First, this was a cross-sectional study of a college student population, and we only analyzed the association between the two factors rather than the causal relationship. The causal relationship between dairy consumption and psychological symptoms should be explored in depth in future research in conjunction with longitudinal studies. Second, the questionnaire in our study was used to recall dairy consumption, psychological symptoms, and other related factors in the past; thus, the results may have been biased because of the influence of individual recall ability. Third, we collected data on the frequency of weekly dairy intake, but not on the total weekly dairy intake and type of dairy products. In previous studies, a higher frequency of low-fat dairy consumption was associated with a lower prevalence of depressive symptoms [41], and full-fat dairy products may be associated with poorer mental health [42]. Different intakes of dairy products and different types of dairy products may have effects of different magnitudes on promoting mental health, and these gaps should be improved in future studies. Our study provides a reference for better dairy consumption by Chinese college students in the future to better promote healthy physical and mental development.

## 5. Conclusions

The results revealed that Chinese college students with lower dairy consumption had a higher detection rate of psychological symptoms. Dairy consumption was negatively associated with the occurrence of psychological symptoms. These findings suggest that the dairy consumption of Chinese college students should be increased in the future. Additionally, dietary health education and psychological health education should be provided to ensure a healthy diet for Chinese college students, while simultaneously reducing their psychological symptoms and promoting healthy physical and mental development.

## Figures and Tables

**Table 1:** Comparison of dairy consumption among college students with different characteristics in China.

Characteristics	Dairy Consumption			Total	*χ*^2^/*F*-Value	*p*-Value
	≤2 Times/Week	3–5 Times/Week	≥6 Times/Week			
N	1516	2485	1903	5904		
Sex						
Male	765 (50.5)	1013 (40.8)	776 (40.8)	2554 (43.3)	43.115	<0.001
Female	751 (49.5)	1472 (59.2)	1127 (59.2)	3350 (56.7)		
SES						
Low	324 (21.4)	370 (14.9)	239 (12.6)	933 (15.8)	87.779	<0.001
Medium	1037 (68.4)	1806 (72.7)	1323 (69.5)	4166 (70.6)		
High	155 (10.2)	309 (12.4)	341 (17.9)	805 (13.6)		
Screen time (h/d),N(%)						
<2 h/d	340 (22.4)	584 (23.5)	584 (30.7)	1508 (25.5)	39.678	<0.001
≥2 h/d	1176 (77.6)	1901 (76.5)	1319 (69.3)	4396 (74.5)		
SSB Consumption						
≤2 times/week	725 (47.8)	1427 (57.4)	1209 (63.5)	3361 (56.9)	87.275	<0.001
3–5 times/week	417 (27.5)	582 (23.4)	389 (20.4)	1388 (23.5)		
≥6 times/week	374 (24.7)	476 (19.2)	305 (16.0)	1155 (19.6)		
Sleep Quality(PSQI)						
Good	348 (23.0)	637 (25.6)	637 (33.5)	1622 (27.5)	54.136	<0.001
Poor	1168 (77.0)	1848 (74.4)	1266 (66.5)	4282 (72.5)		
BMI (kg/m^2^)	23.53 ± 6.17	23.25 ± 6.34	23.28 ± 6.20	23.33 ± 6.25	1.034	0.356
MVPA (min/day)	20.92 ± 16.82	20.59 ± 16.98	24.60 ± 20.49	21.97 ± 18.24	29.821	<0.001

**Table 2:** Comparison of the detection rate of psychological symptoms among college students with different dairy consumption habits in China.

Psychological Symptoms	Dairy Consumption	N	Percentage	*χ^2^*-Value	*p*-Value
Male					
Emotional symptoms	≤2 times/week	174	22.7	24.167	<0.001
	3–5 times/week	152	15.0		
	≥6 times/week	112	14.4		
Behavioral symptoms	≤2 times/week	182	23.8	29.947	<0.001
	3–5 times/week	156	15.4		
	≥6 times/week	110	14.2		
Social adaptation difficulties	≤2 times/week	162	21.2	29.290	<0.001
	3–5 times/week	146	14.4		
	≥6 times/week	89	11.5		
Psychological symptoms	≤2 times/week	168	22.0	29.641	<0.001
	3–5 times/week	146	14.4		
	≥6 times/week	96	12.4		
Female					
Emotional symptoms	≤2 times/week	184	24.5	19.522	<0.001
	3–5 times/week	291	19.8		
	≥6 times/week	183	16.2		
Behavioral symptoms	≤2 times/week	178	23.7	17.899	<0.001
	3–5 times/week	299	20.3		
	≥6 times/week	180	16.0		
Social adaptation difficulties	≤2 times/week	137	18.2	8.610	0.013
	3–5 times/week	220	14.9		
	≥6 times/week	150	13.3		
Psychological symptoms	≤2 times/week	173	23.0	20.461	<0.001
	3–5 times/week	272	18.5		
	≥6 times/week	167	14.8		
Total					
Emotional symptoms	≤2 times/week	358	23.6	38.278	<0.001
	3–5 times/week	443	17.8		
	≥6 times/week	295	15.5		
Behavioral symptoms	≤2 times/week	360	23.7	40.611	<0.001
	3–5 times/week	455	18.3		
	≥6 times/week	290	15.2		
Social adaptation difficulties	≤2 times/week	299	19.7	34.521	<0.001
	3–5 times/week	366	14.7		
	≥6 times/week	239	12.6		
Psychological symptoms	≤2 times/week	341	22.5	45.062	<0.001
	3–5 times/week	418	16.8		
	≥6 times/week	263	13.8		

**Table 3:** Multiple logistic regression analysis of dairy consumption and psychological symptoms among Chinese college students (*n* = 5904).

Psychological Symptoms	Dairy Consumption	Odds Ratio (95% Confidence Interval)		
		Crude Model	Model 1	Model 2
Male				
Emotional symptoms	≥6 times/week	1	1	1
	3–5 times/week	1.05 (0.80, 1.36)	1.04 (0.80, 1.36)	0.75 (0.57, 1.00)
	≤2 times/week	1.75 (1.34, 2.27) ^a^	1.68 (1.29, 2.19) ^a^	1.16 (0.87, 1.54)
	P for trend	<0.001	<0.001	<0.001
Behavioral symptoms	≥6times/week	1	1	1
	3–5 times/week	1.10 (0.85, 1.44)	1.10 (0.84, 1.43)	0.81 (0.61, 1.08)
	≤2 times/week	1.89 (1.46, 2.46) ^a^	1.84 (1.42, 2.40) ^a^	1.28 (0.96, 1.71)
	P for trend	<0.001	<0.001	<0.001
Social adaptation difficulties	≥6 times/week	1	1	1
	3–5 times/week	1.30 (0.98, 1.72)	1.28 (0.97, 1.70)	0.94 (0.69, 1.28)
	≤2 times/week	2.07 (1.57, 2.75) ^a^	2.00 (1.51, 2.66) ^a^	1.37 (1.01, 1.87)
	P for trend	<0.001	<0.001	<0.001
Psychological symptoms	≥6 times/week	1	1	1
	3–5 times/week	1.19 (0.91, 1.57)	1.18 (0.90, 1.56)	0.87 (0.65, 1.17)
	≤2 times/week	1.99 (1.52, 2.62) ^a^	1.91 (1.45, 2.52) ^a^	1.35 (1.01, 1.81)
	P for trend	<0.001	<0.001	<0.001
Female				
Emotional symptoms	≥6 times/week	1	1	1
	3–5 times/week	1.27 (1.04, 1.56)	1.25 (1.02, 1.54)	1.14 (0.92, 1.42)
	≤2 times/week	1.67 (1.33, 2.11) ^a^	1.54 (1.22, 1.95) ^a^	1.38 (1.08, 1.77) ^a^
	P for trend	<0.001	<0.001	<0.001
Behavioral symptoms	≥6 times/week	1	1	1
	3–5 times/week	1.34 (1.09, 1.64)	1.32 (1.07, 1.62)	1.22 (0.98, 1.51)
	≤2 times/week	1.63 (1.30, 2.06) ^a^	1.51 (1.19, 1.91)	1.33 (1.04, 1.69)
	P for trend	<0.001	<0.001	<0.001
Social adaptation difficulties	≥6 times/week	1	1	1
	3–5 times/week	1.15 (0.92, 1.43)	1.11 (0.88, 1.39)	1.03 (0.81, 1.31)
	≤2 times/week	1.45 (1.13, 1.87)	1.32 (1.02, 1.70)	1.08 (0.83, 1.42)
	P for trend	<0.001	<0.001	<0.001
Psychological symptoms	≥6 times/week	1	1	1
	3–5 times/week	1.30 (1.06, 1.61)	1.28 (1.03, 1.58)	1.18 (0.94, 1.47)
	≤2 times/week	1.72 (1.36, 2.18) ^a^	1.57 (1.23, 1.99) ^a^	1.45 (1.13, 1.86)
	P for trend	<0.001	<0.001	<0.001
Total				
Emotional symptoms	≥6 times/week	1	1	1
	3–5 times/week	1.18 (1.01, 1.39)	1.17 (0.99, 1.37)	0.99 (0.84, 1.18)
	≤2 times/week	1.69 (1.42, 2.00) ^a^	1.57 (1.32, 1.87) ^a^	1.29 (1.08, 1.55)
	P for trend	<0.001	<0.001	<0.001
Behavioral symptoms	≥6 times/week	1	1	1
	3–5 times/week	1.25 (1.06, 1.47)	1.23 (1.05, 1.45)	1.06 (0.90, 1.25)
	≤2 times/week	1.73 (1.46, 2.06) ^a^	1.63 (1.37, 1.94) ^a^	1.31 (1.09, 1.57)
	P for trend	<0.001	<0.001	<0.001
Social adaptation difficulties	≥6 times/week	1	1	1
	3–5 times/week	1.20 (1.01, 1.43)	1.18 (0.99, 1.40)	1.00 (0.83, 1.20)
	≤2 times/week	1.71 (1.42, 2.06) ^a^	1.59 (1.32, 1.92) ^a^	1.21 (0.99, 1.48)
	P for trend	<0.001	<0.001	<0.001
Psychological symptoms	≥6 times/week	1	1	1
	3–5 times/week	1.26 (1.07, 1.49)	1.24 (1.05, 1.47)	1.07 (0.90, 1.27)
	≤2 times/week	1.81 (1.52, 2.16) ^a^	1.68 (1.40, 2.01) ^a^	1.42 (1.18, 1.71) ^a^
	P for trend	<0.001	<0.001	<0.001

Note: Model 1 controlled for age, SES; Model 2 controlled for screen time, sugar-sweetened beverages, and sleep quality on the basis of Model 1, BMI, MVPA. ^a^, *p* < 0.001.

## Data Availability

To protect the privacy of participants, the questionnaire data will not be disclosed to the public. If necessary, the corresponding author can be contacted.
